# Influential Mechanism of Natural Organic Matters with Calcium Ion on the Anion Exchange Membrane Fouling Behavior via xDLVO Theory

**DOI:** 10.3390/membranes11120968

**Published:** 2021-12-09

**Authors:** Zhun Ma, Lu Zhang, Ying Liu, Xiaosheng Ji, Yuting Xu, Qun Wang, Yongchao Sun, Xiaomeng Wang, Jian Wang, Jianliang Xue, Xueli Gao

**Affiliations:** 1 College of Chemical and Biological Engineering, Shandong University of Science and Technology, Qingdao 266590, China; skdmaz919@sdust.edu.cn (Z.M.); luluzh5709@163.com (L.Z.); ly19980407@163.com (Y.L.); xyt15153393708@163.com (Y.X.); yongchao_sun@163.com (Y.S.); 15764237673@163.com (X.W.); 2 Sanya Institute of Oceanology, Chinese Academy of Sciences, Sanya 572000, China; 3 Key Laboratory of Marine Chemistry Theory and Technology, Ministry of Education, College of Chemistry and Chemical Engineering, Ocean University of China, Qingdao 266100, China; gxl_ouc@126.com; 4 The Institute of Seawater Desalination and Multipurpose Utilization, Ministry of Natural Resources (MNR), Tianjin 300192, China; swordking8856@163.com; 5 College of Safety and Environmental Engineering, Shandong University of Science and Technology, Qingdao 266590, China; ll-1382@163.com

**Keywords:** membrane fouling, xDLVO theory, natural organic matters, anion exchange membrane

## Abstract

The fouling mechanism of the anion exchange membrane (AEM) induced by natural organic matter (NOM) in the absence and presence of calcium ions was systematically investigated via the extended Derjaguin–Landau–Verwey–Overbeek (xDLVO) approach. Sodium alginate (SA), humic acid (HA), and bovine serum albumin (BSA) were utilized as model NOM fractions. The results indicated that the presence of calcium ions tremendously aggravated the NOM fouling on the anion exchange membrane because of Ca-NOM complex formation. Furthermore, analysis of the interaction energy between the membrane surface and foulants via xDLVO revealed that short-range acid–base (AB) interaction energy played a significant role in the compositions of interaction energy during the electrodialysis (ED) process. The influence of NOM fractions in the presence of calcium ions on membrane fouling followed the order: SA > BSA > HA. This study demonstrated that the interaction energy was a dominating indicator for evaluating the tendency of anion exchange membranes fouling by natural organic matter.

## 1. Introduction

Electrodialysis (ED) has been not only extensively employed for the desalination of seawater and brackish water but also actively utilized for the reclamation of high-salinity industrial wastewater [1,2,3,4,5,6,7,8]. However, a serious impediment in operation of the ED process is ion-exchange membranes (IEMs) fouling, which would increase the electric resistance of the membrane and decrease current efficiency, resulting in deterioration of the electrodialysis performance [9,10,11,12,13]. Many studies have indicated that the natural organic matter (NOM) played a crucial role in ion exchange membranes fouling, especially the anion exchange membranes (AEMs) fouling [14,15,16]. In general, the AEMs fouling was attributed to the electrostatic interaction between the NOM and fixed positive charged functional groups of AEMs [17,18,19]. In addition, the AEMs were susceptible to be fouled due to hydrophilic/hydrophobic interaction and geometrical factors containing the chemical structure of foulants and surface morphology of AEMs [20,21]. Furthermore, several studies have found that inorganic ions can change organic material characteristics, affecting the attachment of NOM to the membrane surface [22,23,24]. As a common inorganic ion, calcium ions easily react with the surface of organic molecules and form calcium–carboxylate complex formation, which would aggravate membrane organic fouling [25,26].

Membrane fouling, essentially attributed to interface interactions between the membrane surface and foulants in aqueous solution, is a big obstacle to the ED process for wastewater treatment [27]. Therefore, analysis of these interface interactions may give new insight to understanding the mechanism of membrane fouling. The xDLVO theory is usually used to interpret these interactions and then to investigate the membrane fouling mechanism [28,29]. Moreover, this theory is a method for quantitatively studying the interfacial interaction between the membrane surface and foulants (Lishitz-van der waals (LW), electrostatic (EL), and short-path acid–base (AB) interaction energy), which can effectively predict the trend of membrane fouling. Lin et al. indicated that the xDLVO theory could verify the impacts of different components of organic matter on ultrafiltration membrane fouling [30]. Furthermore, Lin et al. employed the xDLVO theory to predict the organic membranes fouling in ultrafiltration systems, and the simulation results were in agreement with the experimental results [31]. Kim et al. used interfacial forces based on xDLVO to elucidate membrane fouling in the RO process [32]. Shan et al. used HA as a model foulant to study its fouling behavior on super-wetting nanofiltration membranes applying xDLVO approach. They testified that the super-hydrophilic membrane had the strongest repulsion force to HA due to the highest positive total interaction energy value [27]. Zhao et al. utilized xDLVO theory to quantitatively validate the interfacial interactions in the nanofiltration membrane fouling process under various organic matter and Ca^2+^ concentration [33]. However, to the best of our knowledge, there are no reports on the application of xDLVO theory to predict and evaluate anion exchange membrane fouling in the ED process. Consequently, it is very important to investigate the membrane–foulants interaction energy to give an insight into the fouling mechanism and to develop effective prevention strategies of membrane fouling in the application of ED.

The motivation of the study was to explore the fouling mechanism of natural organic matters combined with or without calcium ions on AEMs. The interaction energy was calculated by xDLVO theory to elucidate how the behavior of calcium ions aggravated the organic AEMs fouling during the ED process. The membrane surface properties were investigated by contact angle, transform infrared spectroscopy (FTIR), and scanning electron microscope (SEM) in order to verify the feasibility of xDLVO theory in predicting AEMs fouling during the ED process. Experimental results were expected to comprehend the AEMs fouling mechanisms of natural organic matter combined with calcium ions during the ED process via xDLVO theory.

## 2. Theory

The interaction energy calculated by xDLVO, membrane–foulants interaction energy in aqueous media, is the summation of LW, AB, and EL interactions, as follows [29]:

(1) $$U_{\mathrm{mlf}}^{\mathrm{XDLVO}}=U_{\mathrm{mlf}}^{\mathrm{EL}}+U_{\mathrm{mlf}}^{\mathrm{LW}}+U_{\mathrm{mlf}}^{\mathrm{AB}}$$

where $U_{\mathrm{mlf}}^{\mathrm{XDLVO}}$(mJ/m^2^) is the total interaction energy between the foulants and membrane surface, $U_{\mathrm{mlf}}^{\mathrm{EL}}$(mJ/m^2^) is the EL interaction energy, $U_{\mathrm{mlf}}^{\mathrm{LW}}$(mJ/m^2^) is the LW interaction energy, and $U_{\mathrm{mlf}}^{\mathrm{AB}}$(mJ/m^2^) is the AB interaction energy. In addition, “m”, “l”, and “f” denote the membrane, water, and foulants, respectively.

Correspondingly,$\;U_{\mathrm{mlf}}^{\mathrm{LW}}$,$\;U_{\mathrm{mlf}}^{\mathrm{AB}}$, and$\;U_{\mathrm{mlf}}^{\mathrm{EL}}$ can be expressed as,

(2) $$U_{\mathrm{mlf}}^{\mathrm{LW}}\left(y\right)=2\pi \Delta G_{y_{0}}^{\mathrm{LW}}\frac{y_{0}^{2}a_{c}}{y}$$

(3) $$U_{\mathrm{mlf}}^{\mathrm{AB}}\left(y\right)=2\pi a_{c}\lambda \Delta G_{y_{0}}^{\mathrm{AB}}\exp\left[\frac{y_{0}-y}{\lambda }\right]$$

(4) $$U_{\mathrm{mlf}}^{\mathrm{EL}}\left(y\right)=\pi \varepsilon_{0}\varepsilon_{r}a_{c}\left[2\zeta_{f}\zeta_{m}ln\left(\frac{1+e^{-\kappa y}}{1-e^{-\kappa y}}\right)+\left(\zeta_{f}^{2}+\zeta_{m}^{2}\right)\ln\left(1-e^{-2\kappa y}\right)\right]$$

where y(nm) is the separation distance between the foulants and membrane surface, ε_γ_ is the relative dielectric constant, C^2^/(N·m^2^); ε_0_ is the vacuum dielectric constant, C^2^/(N·m^2^); $y_{0}$ is the minimum separation distance between interaction surfaces (usually taken as 0.158 nm); ζ is the zeta potential; κ is the inverse Debye screening length (0.104 nm^−1^); and λ is the decay length of the AB interaction in water (0.6 nm).

The interfacial free energy$\;\Delta G_{y_{0}}^{\mathrm{EL}}$,$\;\Delta G_{y_{0}}^{\mathrm{LW}}$, and$\;\Delta G_{y_{0}}^{\mathrm{AB}}$ are the interaction energy per unit area of EL, LW, and AB, respectively, as expressed in mJ/m^2^, which can be obtained as follows:

(5) $$\Delta G_{y_{0}}^{\mathrm{LW}}=-2\left(\sqrt{\gamma_{m}^{\mathrm{LW}}}-\sqrt{\gamma_{l}^{\mathrm{LW}}}\right)\left(\sqrt{\gamma_{f}^{\mathrm{LW}}}-\sqrt{\gamma_{l}^{\mathrm{LW}}}\right)$$

(6) $$\Delta G_{y_{0}}^{\mathrm{AB}}=2\left[\sqrt{\gamma_{l}^{+}}\left(\sqrt{\gamma_{f}^{-}}+\sqrt{\gamma_{m}^{-}}-\sqrt{\gamma_{l}^{-}}\right)+\sqrt{\gamma_{l}^{-}}\left(\sqrt{\gamma_{f}^{+}}+\sqrt{\gamma_{m}^{+}}-\sqrt{\gamma_{l}^{+}}\right)-\left(\sqrt{\gamma_{f}^{-}\gamma_{m}^{+}}+\sqrt{\gamma_{f}^{+}\gamma_{m}^{-}}\right)\right]$$

where $\gamma^{\mathrm{LW}}$ is the van der Waals surface tension component, and$\;\gamma^{+}$ and $\gamma^{-}$ are the electron acceptor surface tension component and the electron donor surface tension component, respectively, as expressed in mJ/m^2^.

The surface tension parameters ($\gamma_{S}^{\mathrm{LW}}$, $\gamma_{S}^{+}$, and $\gamma_{S}^{-}$) for the membranes and foulants can be calculated by the extended Young equation given by

(7) $$\left(\gamma_{L}^{\mathrm{LW}}+2\sqrt{\gamma_{L}^{+}\gamma_{L}^{-}}\right)\left(1+\cos\theta \right)=2\left(\sqrt{\gamma_{S}^{\mathrm{LW}}\gamma_{L}^{\mathrm{LW}}}+\sqrt{\gamma_{S}^{+}\gamma_{L}^{-}}+\sqrt{\gamma_{S}^{-}\gamma_{L}^{+}}\right)$$

where θ is the contact angle, and${\;\gamma }_{L}^{\mathrm{LW}}$,$\gamma_{L}^{+}$, and $\gamma_{L}^{-}$ are the known surface tension properties for three probe liquids.

The total surface tension $\gamma^{\mathrm{TOT}}$(mJ/m^2^) and the AB components of surface tension (mJ/m^2^) are expressed as follows:

(8) $$\gamma^{\mathrm{TOT}}=\gamma^{\mathrm{AB}}+\gamma^{\mathrm{LW}}$$

(9) $$\gamma^{\mathrm{AB}}=2\sqrt{\gamma^{+}\gamma^{-}}.$$

## 3. Materials and Methods

### 3.1. Materials

Commercial homogeneous AEMs (SELEMION AMV) and CEMs (SELEMION CMV) were provided by AGC ENGINEERING CO., LTD (Chiba, Japan). The detailed properties of the ion exchange membrane are presented in Table S1. Three natural organic matters such as SA, HA, and BSA were utilized as model foulants and purchased from Sinopharm Chemical Reagent Co., Ltd., Shanghai, China. The stock solutions (0.1 g/L SA, 0.1 g/L HA, and 0.1 g/L BSA) were prepared by dissolving the SA, HA, and BSA in pure water, and they had to be mixed for 24 h to ensure the complete dissolution of foulants. After that, the stock solutions were filtered by glass–fiber membrane (0.45 μm, GF/F, Whatman, UK). The filtered stock solutions were placed in the refrigerator at 4 °C. Reagent grade salt of NaCl and CaCl_2_ (Sinopharm Chemical Reagent Co., Ltd., Shanghai, China) were used to prepare the solutions for fouling experiments. The electrode rinse solution was prepared by adding Na_2_SO_4_ (AR, Sinopharm Chemical Reagent Co., Ltd., Shanghai, China). All solutions were prepared by pure water, which was provided by one standalone water purification system (Yantai Huiquan Equipment Co., Ltd., Yantai, China).

### 3.2. Experimental Protocol

The self-made bench-scale ED apparatus was utilized as presented schematically in Figure S1. It consisted of a membrane stack, a DC power supply (PS-305DM, Longwei Struments (HK) Co., Ltd., Hong Kong, China), and four peristaltic pumps (CXB-30, Wenzhou Erle Pump Co., Ltd., Wenzhou, China). The membrane stack consisted of dilute, concentrated, and electrode compartments, which were separated by three pristine CEMs and two pristine AEMs. The DC power supply was connected to membrane stack through two ruthenium-coated titanium electrodes. The effective area of AEM and CEMs was all 9 cm^2^ in this work.

During fouling experiments, the 800 mL initial solution was composed of 10 mmol/L NaCl and different foulants (as shown in Table S2) in the dilute compartment and the concentrated compartment. The two compartments were circulated by peristaltic pumps at a flow rate of 150 mL/min, respectively. The electrode rinse solution was 800 mL of 0.01 mol/L Na_2_SO_4_. Fouling experiments were performed for a continuous period of 20 h at ambient temperature (20 ± 0.5 °C). The observation of membrane fouling was carried out under a constant voltage of 3.0 V. Moreover, the conductivity of the dilute compartment solution was measured every 30 min using a conductivity meter (DDS-307, Shanghai INESA Scientific Instrument Co., Ltd., Shanghai, China). All of the fouling experiments were carried out three times, and the data shown in the paper were the average of the three experiments. It should be noted that the ratios of the fluctuating value to the average were less than 0.1%; thus, the error bars were not added in the figures and tables. After fouling experiments, the used AEMs were rinsed using pure water and then dried naturally or submerged in pure water for further characterization.

### 3.3. Characterization Method

Morphology of the original and used membranes were observed by SEM (Hitachi S4800, Tokyo, Japan) at an accelerating voltage of 10 kV and the magnification of 1.00 k. The chemical compositions of membranes were determined using ATR-FTIR Spectrometer (Nicolet 380, Madison, WI, USA). A spectrum collected as the average of 32 scans with a resolution of 4 cm^−1^ was recorded from 4000 to 400 cm^−1^. A contact angle meter (Kruss DSA30, Hamburg, Germany) was utilized to measure the contact angles of three probe liquids including ultrapure water, glycerol, and diiodomethane on the original and used membrane surfaces, and the sessile drop method was used in all measurements. The zeta potentials of the original and used AEMs were examined by the zeta potential analyzer (Zeta 90 Plus, Brookhaven Instruments, New York, USA). The zeta potential and particle size distribution of organic colloid were determined by dynamic light scattering with a Zetasizer Nano S90 (ZEN1690, Malvern instruments Ltd., Malvern, UK). For each sample, at least triplicate measurements were carried out in order to ensure the accuracy, and the average values were used in this work. Membranes for SEM, FTIR, and contact angle were dried at 38 °C for 24 h before measurements.

## 4. Results and Discussion

### 4.1. Influence of SA with Calcium Ions on Membrane Fouling

Recent studies had demonstrated that divalent cations such as Ca^2+^ had a dramatic effect on SA fouling in the membrane process [34,35]. In the current work, SA with the presence of Ca^2+^ had been employed as a model foulant to investigate organic fouling in the ED process. Table 1 showed the particle zeta potential, colloid sizes, and interfacial interaction energy in different membrane–SA–calcium ion systems. It could be seen that variations in the interfacial interaction energy were illustrated in different membrane–SA–calcium ion systems, due to differences in Ca^2+^ concentration. In terms of the xDLVO theory, a positive value for the total interaction energy between membrane and foulants indicated the resistance to membrane fouling, while a negative value means a promotion to membrane fouling [36,37]. In all membrane–SA–calcium ion systems, the negative value of the total interaction energy could be inferred that the foulant of SA tended to exacerbate the membrane fouling. Lower interaction energy occurred when calcium ion was absent in the aqueous solution. This demonstrated that calcium ions exhibited a predominant contribution to AEMs fouling. Simultaneously, the total interaction energy between SA and anion exchange membrane firstly increased and then decreased with the increase in calcium ion concentration. Similar results were published previously [38,39] and could be explicated by charge neutralization as well as the SA–calcium ionic bridge that formed, which decreased the electrostatic repulsion and increased the attractive energy between SA molecules and the membrane surface. Hence, the total interaction energy changed from −7.7383 to −22.0968 mJ/m^2^, revealing that calcium ions had a very great influence on the membrane fouling by SA.

As could be seen in Table 1, the particle size of SA increased as the calcium ions increased. The particle size was the smallest in SA solutions without calcium ions, while the maximum particle size occurred at a calcium ion concentration of 8 mmol. It was observed that calcium ions could enlarge the particle size of SA due to carboxylic functional groups in SA molecules, which bound them together via calcium ion bridging, leading to the formation of macromolecular chelate [40,41]. During the electrodialysis process, the ionic bridge among SA and between SA and the membrane surface with the presence of Ca^2+^ facilitated the formation of an across-linked organic gel layer on the AEMs surface [42].

The zeta potential is an important parameter that governs the electrostatic interaction between the organic compound and membrane. The addition of calcium ions in aqueous solutions increased the particle zeta potential, and the zeta potential increased with the increasing calcium ion concentration. The shielding effect of calcium ions and complexation of calcium ions with SA functional groups can effectively neutralize the electronegativity of alginate [43], resulting in the particle zeta potential increasing. The zeta potential would reflect the electrostatic forces of mutual exclusion or attraction between particles. The particles tend to condense or agglomerate at a lower zeta potential value (positive or negative) because the attraction exceeds the repulsive force. The important utilization of zeta potential is to investigate the interaction of colloids with electrolytes due to many charged colloids interact with the electrolyte in a complicated manner. In this study, positively charged calcium ions reduced the absolute value of zeta potential and promoted the agglomeration of SA molecules, leading to membrane fouling aggravation. Furthermore, SA would reduce AEMs’ hydrophilicity and increase hydrophobicity, which represents an attractive effect to exacerbate membrane fouling [15,44,45].

**Figure 1:**
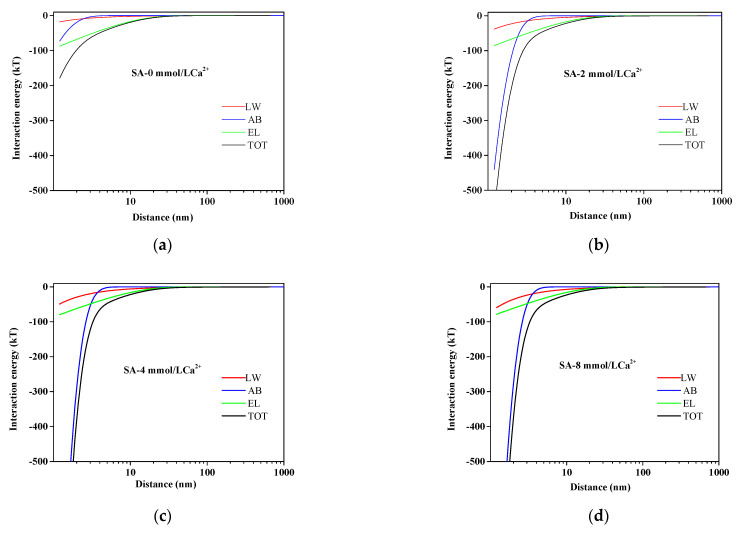
Variation of AEM-SA interaction energy profiles at different calcium ion concentrations: (**a**) SA without Ca^2+^; (**b**) SA with 2 mmol/L Ca^2+^; (**c**) SA with 4 mmol/L Ca^2+^; (**d**) SA with 8 mmol/L Ca^2+^.

Figure 1 presented the variation of AEM–SA interaction energy with separation distances between the surface of AEM and SA in different calcium ion concentration solutions. It could be seen that the interaction energy including TOT, LW, AB, and EL gradually closed to a zero value with the increasing of the distance between SA molecules and AEM. The results revealed that the attractive reaction had gradually enhanced, which led to an aggravation of membrane fouling in the presence of calcium ions. As seen in Figure 1, the total interaction energy curve was steepest at 4 mmol/L calcium ion concentration. It was inferred that the U^AB^ made the dominant contribution to the U^TOT^ (corresponding to U^xDLVO^) when the separation distance was less than 5 nm with the presence of calcium ions. Among them, AB was mostly affected by calcium ions; it is typical for Lewis acid to act as an electron acceptor. Moreover, calcium ions would increase the surface tension parameters of the Lewis acid/base further to increase the magnitude of U^AB^ determined by the surface tension parameters of the Lewis acid/base, which was proven in the previously reported study [46,47,48].

**Figure 2:**
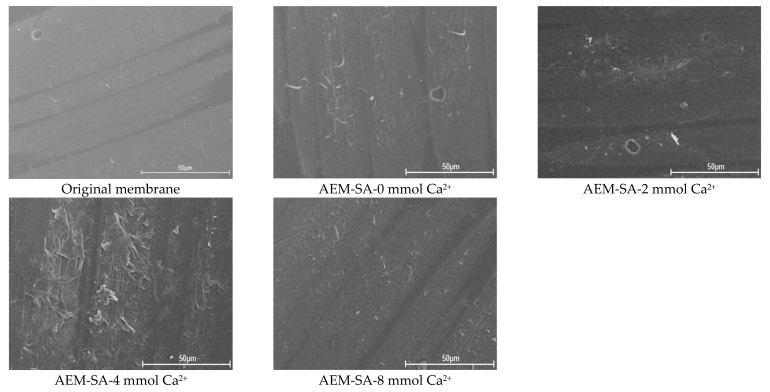
SEM images of original and used AEMs in the AEM-SA system with different calcium ion concentrations.

The FTIR spectrum and SEM images of the original and used membrane are shown in Figure 2 and Figure S2. From the FTIR spectrum of the virgin membrane, it could be that the peak at 3300–3100 cm^−1^ was a specific peak of C-H in the benzene ring; and the peaks around 1580 cm^−1^ and 1480 cm^−1^ are characteristic absorption peaks of the benzene ring skeleton; besides, there is a peak around 2923 cm^−1^, which was the peak of -CH_3_. For the membrane after SA with Ca^2+^ fouling, there is a distinct broad peak at 3500–3100 cm^−1^ in the FTIR spectrum. Compared with the original membrane, this peak shape was wider. This was the effect of the influence of the stretching vibration of the associated -OH. A new characteristic peak occurred at around 1650 cm^−1^, which corresponded to a C=O bond. The peak of the C-H bond was located near 2924 cm^−1^. It indicated the adhesion of SA on the anion exchange membrane. Additionally, with the presence of a calcium ion, the strong foulant–foulant attractive interaction would accelerate the formation of a compact gel layer of SA on the membrane surface, which resulted in the more severe fouling of AEMs. This was verified by the SEM images of the membrane surface, as shown in Figure 2. For example, the membrane surfaces were contaminated by foulants and gradually formed a dense gel layer with the absence and presence of calcium ion (Figure 2).

### 4.2. Influence of HA with Calcium Ions on Membrane Fouling

In the case of HA with the absence and presence of Ca^2+^ fouling, the variation of the particle zeta potential, the colloid size, and the interfacial energy between HA and the anion exchange membrane are presented in Table 2. It could be seen that calcium ions have made a difference in the AEM–HA interaction energy. Based on the xDLVO theory, the presence of calcium ions could deteriorate membrane fouling. Moreover, the membrane fouling of HA to AEM was aggravated as the Ca^2+^ concentrations increased, and the most serious fouling of AEM occurred at a calcium ion concentration of 8 mmol/L.

As shown in Table 2, changes in the zeta potential and colloidal size of HA particles indicated that the concentration of calcium ions had a dominant effect on HA characteristics because HA had a more negative charge than SA. Zeta potentials varied from −55.66 mV to −20.07 mV with the increasing Ca^2+^ concentrations because the complexation of carboxyl groups with Ca^2+^ neutralized the electronegativity of HAs. In addition, the colloid size of HA changed from 285.9 to 596.4 nm due to the reducing of electrostatic repulsion between HA molecules, which was conducive to the aggregation of HA colloidal particles. Moreover, the bridging between Ca^2+^ and HA macromolecules accelerates the increasing colloid size of HA particles, which were liable to adhere on the surface of AEMs, deteriorating membrane fouling.

**Figure 3:**
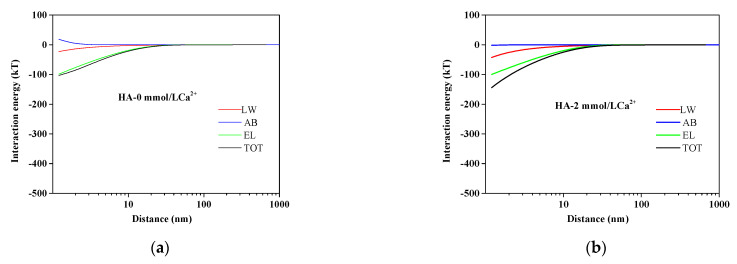

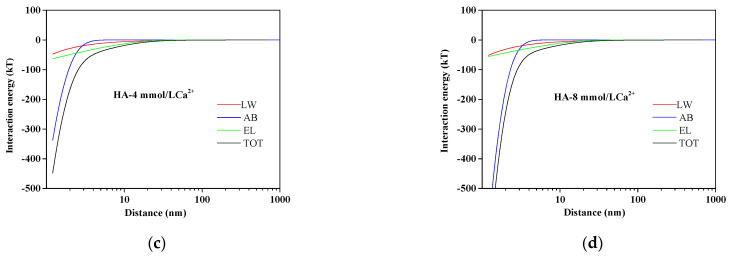
Variation of AEM–HA interaction energy profiles at different calcium ion concentrations: (**a**) HA without Ca^2+^; (**b**) HA with 2 mmol/L Ca^2+^; (**c**) HA with 4 mmol/L Ca^2+^; (**d**) HA with 8 mmol/L Ca^2+^.

The HA fouling process was determined by the mutual interactions between AEM and HA and among HA molecules. The quantitative evaluations of these interactions could be acquired by the xDLVO theory. The calculated total interaction energy with different Ca^2+^ concentrations is also presented in Table 2. It could be observed that the total interaction energy was enhanced from −4.3385 to −18.5625 mJ/m^2^ with increasing Ca^2+^ concentrations, indicating that Ca^2+^ played a significant role in the total interaction energy. To further explore the influencing mechanism of interactions on membrane fouling, the interaction energy at different separation distances between the membrane surface and HA in different Ca^2+^ concentrations is illustrated in Figure 3. As shown in Figure 3, the positive AB interaction energy increased slightly as the distance of HA to AEM decreased in the absence of Ca^2+^. Nevertheless, the values of LW, EL, and TOT interaction energy were all negative and had minor variations as the distance of HA to AEM decreased without Ca^2+^. With the addition of calcium ions, the value of AB became negative, and its curve became steeper with the calcium ions concentration increasing. The TOT interaction energy curve trend was similar to that of AB. It could be revealed that the AB interaction energy accounted for the main proportion of total interaction energy, while the interaction energy of LW and EL had a small impact on the energy of TOT interaction. The negative interaction energy decreased as the distance of HA to AEM decreased in the presence of Ca^2+^, which indicated an adhesive reaction enhanced between HA and AEMs leading to severe membrane fouling.

Comparing the FTIR spectra of original and the used membrane by HA together with 4 mmol Ca^2+^, as shown in Figure S3, a broad adsorption peak at 3400 cm^−1^ was attributed to the overlapping of bands from the stretching vibrations of N-H and O-H, which revealed the presence of HA. Moreover, the characteristic bands of HA such as C = O stretching vibration, C-N stretching vibration, and C-O stretching vibration were assigned at 1610 cm^−1^, 1444 cm^−1^, and 1093 cm^−1^, respectively. Meanwhile, the representative bands of used AEM such as C-H bending vibration at 1376 cm^−1^ respond at a lower wavenumber. A lower wavenumber denotes the increase in the bond length, and this might be attributed to the electrostatic and affinity interaction between HA and AEM [18,49,50].

**Figure 4:**
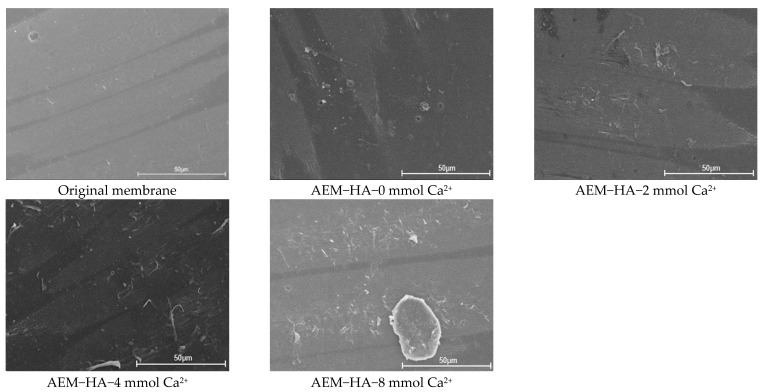
SEM images of original and used AEMs in AEM-HA system with different calcium ion concentration.

Figure 4 presented the surface morphology of the different used AEMs in feed solutions containing 0.1 g/L HA with and without Ca^2+^. Comparison of these images indicated that the concentration of Ca^2+^ played a significant role in AEMs fouling. With the ED process performed, more HA–Ca^2+^ complex generated and accumulated onto the AEMs surface, which could form a denser gel layer on the AEMs surface. It could be verified that the charge and hydrophilic characteristics of HA was reduced significantly due to the HA–Ca^2+^ complex formation in the presence of Ca^2+^. However, regardless of the particle size or total interaction energy, the contribution of Ca^2+^ in HA to AEM fouling was less than that of SA. It was revealed that Ca^2+^ combines specifically with carboxylic groups of SA as well as serving as a bridge between adjacent SA molecules, resulting in a more compact gel layer than those formed in the case of HA [47,50,51].

### 4.3. Influence of BSA with Calcium Ions on Membrane Fouling

Variations in the interaction energy of each AEM-BSA-Ca^2+^ system are summarized in Table 3. The total interaction energy in different AEM-BSA-Ca^2+^ systems had negative value, indicating a higher attractive effect. As illustrated in Table 3, Ca^2+^ concentration played a dominant role in the total interaction energy, zeta potential, and colloid size in case of BSA. The total interaction energy decreased from −11.2046 to −20.5718 mJ/m^2^ as the Ca^2+^ concentration increased. However, compared with SA and HA, the influence of Ca^2+^ concentrations on the total interaction energy, zeta potential, and colloid size became less substantial in different AEM–BSA–Ca^2+^ systems. It was possible that BSA molecules have a lower density of -COO^-^ functional groups and thus have a weaker calcium ion bridging effect. In accordance with our study, Gao et al. also discovered the inconspicuous complexation of BSA with calcium ions [52]. Moreover, the size of the BSA colloid increased slightly with increasing Ca^2+^ concentrations, only increased from 364.5 to 548.5 nm. It was further inferred that there was an insignificant bridging effect between BSA molecules and calcium ions. Additionally, the zeta potential of particles could influence membrane fouling, which was dependent on the total interaction energy. As shown in Table 3, the zeta potential of particles increased with increasing Ca^2+^ concentrations. It was observed that with the increase in Ca^2+^ concentrations, the compression of the electric double layer because of the charge shielding effect reduced the electrostatic repulsion among BSA molecules. This was beneficial to the coagulation between BSA molecules and accelerated the formation of a gel layer on the AEMs surface. Nevertheless, the experiment results presented in Table 3 indicated that the effect of BSA colloid size on membrane fouling is more significant than that of the zeta potential, which could be inferred from Equations (2)–(4).

**Figure 5:**
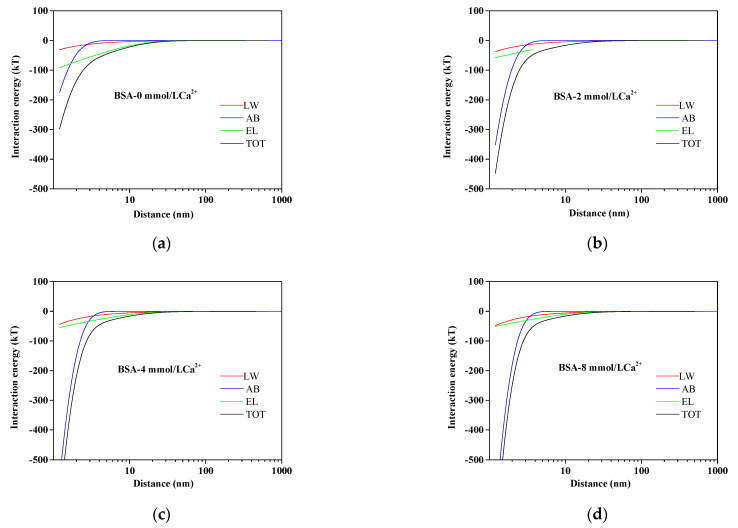
Variation of AEM–BSA interaction energy profiles at different calcium ion concentrations: (**a**) BSA without Ca^2+^; (**b**) BSA with 2 mmol/L Ca^2+^; (**c**) BSA with 4 mmol/L Ca^2+^; (**d**) BSA with 8 mmol/L Ca^2+^.

The results of interaction energy profiles at different Ca^2+^ concentrations are illustrated in Figure 5. It is well known that membrane fouling relied on the total interaction energy, including AB, LW, and EL interaction energy. With the increase in Ca^2+^ concentrations, the TOT interaction energy curve became steeper, indicating that the adhesive reaction became stronger, thus aggravating membrane fouling. Figure 5 also clearly manifested that the value of U^AB^ approached zero when the distance from the BSA particles to the membrane surface was greater than 10 nm, but the U^AB^ played a predominant role in short-distance separation. Therefore, the reduction of U^AB^ was beneficial to alleviate membrane fouling.

**Figure 6:**
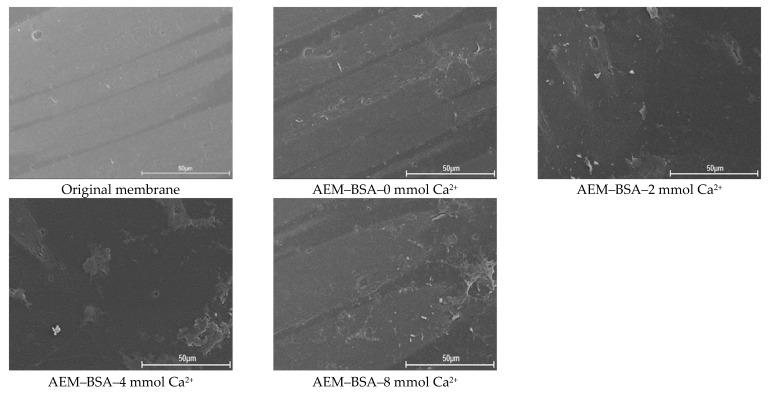
SEM images of original and used AEMs in the AEM–SA system with different calcium ion concentration.

The FITR and SEM were employed to characterize AEMs fouled by the different BSA–Ca^2+^ systems (Figure 6 and Figure S4). As shown in Figure S4, the absorption peak near 1720 cm^−1^ marked the presence of C=O in the amino compound for the protein-like materials. Additionally, the characteristic peaks of 1656 cm^−1^ and 1643 cm^−1^ were observed on the BSA fouled membranes surface, indicating the presence of amide I. Moreover, the peak at 1535 cm^−1^ was exhibited in the FTIR spectra of the BSA fouled membrane, which was attributed to the presence of amide II. As seen from Figure 6, the BSA foulant layer was relatively loose, and the membrane fouling in case of BSA aggravated slightly as the concentration of Ca^2+^ increased compared with that of SA and HA. It was because the BSA molecules interacted with Ca^2+^; unlike SA and HA molecules, it was difficult to generate a gel-type fouling layer on the AEM surface [53]. The gel layer forming of SA resulted from the stronger attractive energy and weaker repulsive energy with SA than that of BSA, where the intermolecular interaction needed to be strong enough to maintain the integrity of the gel layer structure. It could be concluded that there was a much slighter difference of decreased attractive AB energy and LW energy with the increasing of Ca^2+^ concentrations in the case of BSA than that of SA. The results indicated that the interaction energy is a significant factor for evaluating the tendency of AEMs fouling by natural organic matter.

## 5. Conclusions

In the present study, the organic fouling mechanism of AEMs in the ED process was elucidated by xDLVO theory, using SA, HA, and BSA with and without Ca^2+^ as model foulants. The contributions of LW interactions, EL interactions, as well as AB interactions to the total interaction energy that resulted from NOM fractions attaching to the AEMs surface were evaluated. It was worth noting that the short-range acid–base interactions accounted for a more predominating contribution than the van der Waals interactions to the total interaction energy, which played a dominant role in membrane fouling. However, the influence of electrostatic interactions on AEM fouling was negligible due to the slight contribution to the total interaction energy. Meanwhile, ionic bridging between NOM components with the presence of calcium ions resulted in the formation of a complex on the membrane surface, which produced a compact gel layer and tremendously deteriorate anion exchange membrane fouling. These results revealed that the xDLVO theory was a promising strategy to predict and elucidate the NOM fouling of AEMs during the ED process.

## Figures and Tables

**Table 1:** Interaction energy, particle zeta potential, and colloid size in different AEM–SA–Ca^2+^ systems.

[Ca^2+^] /(mmol)	△G^LW^	△G^AB^	△G^EL^	△G^TOT^	Particle Zeta Potential/mV	Colloid Size/nm
0	−4.2392 ^a^	−3.3704	−0.1287	−7.7383	−50.45	264.2
2	−4.6799	−10.6036	−0.0675	−15.3510	−28.9	507.3
4	−4.0827	−17.9737	−0.0404	−22.0968	−21.96	744.6
8	−4.4170	−15.3426	−0.0358	−19.7954	−17.54	832.8

^a^ Interaction energy per unit (mJ/m^2^).

**Table 2:** Interaction energy, particle zeta potential, and colloid size indifferent AEM-HA-Ca^2+^ systems.

[Ca^2+^] /(mmol)	△G^LW^	△G^AB^	△G^EL^	△G^TOT^	Particle Zeta Potential/mV	Colloid Size/nm
0	−5.0024	0.8008	−0.1369	−4.3385	−55.66	285.9
2	−5.6135	−0.0421	−0.0812	−5.7368	−38.12	472.5
4	−5.5391	−7.7161	−0.0449	−13.3001	−24.75	533.3
8	−5.4103	−13.1170	−0.0352	−18.5625	−20.07	596.4

**Table 3:** Interaction energy, particle zeta potential, and colloid size-indifferent AEM–BSA–Ca^2+^ systems.

[Ca^2+^] /(mmol)	△G^LW^	△G^AB^	△G^EL^	△G^TOT^	Particle Zeta Potential/mV	Colloid Size/nm
0	−5.2535	−5.8564	−0.0969	−11.2046	−42.65	364.5
2	−5.3028	−9.9250	−0.0508	−15.2786	−25.31	432.7
4	−5.3231	−13.5620	−0.0403	−18.9254	−21.43	510.2
8	−5.4067	−15.1302	−0.0349	−20.5718	−19.92	548.5

## Supplementary Materials

The following are available online at https://www.mdpi.com/article/10.3390/membranes11120968/s1, Figure S1: The schematic diagram of the experiment; Figure S2: FTIR spectrum of virgin membrane and fouled membrane by SA together with 4 mmol Ca^2+^; Figure S3: FTIR spectrum of fouled membrane by HA together with 4 mmol Ca^2+^; Figure S4: FTIR spectrum of fouled membrane by BSA together with 4 mmol Ca^2+^; Table S1: Properties of ion exchange membranes; Table S2: Concentration of foulants used in the fouling experiments in ED process.

## Nomenclature

y	closest distance between a particle and a planar surface (nm)
a_c_	radius of foulant particle (nm)
e	electron charge (1.6 × 10^−19^ C)
k	Boltzmann’s constant (1.38 × 10^−23^ J K^−1^)
U	interaction energy between the colloid and membrane surface (mJ/m^−2^)
△G	interaction energy per unit area (mJ/m^−2^)
SA	sodium alginate
BSA	bovine serum albumin
Greek letters	
ε_0_	vacuum dielectric constant, C^2^/(*N*·m^2^)
ε_r_	relative dielectric constant, C^2^/(*N*·m^2^)
γ	surface tension parameter (mJ/m^−2^)
ζ	zeta potential (mV)
κ	inverse Debye screening length (0.104 nm^−1^)
λ	decay length of AB interaction in water (0.6 nm)
Superscripts	
AB	short-ranged acid–base
EL	electrostatic
LW	van der Waals
TOT	total
+	electron acceptor
−	electron donor
Subscripts	
f	foulant particle
l	liquid
m	membrane
y_0_	closest separation distance between interaction surfaces (0.158 nm)
