# Supplementary material for: Influential Mechanism of Natural Organic Matters with Calcium Ion on the Anion Exchange Membrane Fouling Behavior via xDLVO Theory
Source: Membranes (Basel). 2021 Dec 9;11(12):968. doi: 10.3390/membranes11120968 (PMC8706472; doi:10.3390/membranes11120968)
Supplement: Supplementary file 1 [file membranes-11-00968-s001.zip › membranes-1474133-supplementary.pdf]

membranes

MDPI

Supplementary Materials: Influential Mechanism of Natural Organic Matters with Calcium Ion on the Anion Exchange Membrane Fouling Behavior via xDLVO Theory

Zhun Ma [1], Lu Zhang [1], Ying Liu [1], Xiaosheng Ji [2,*], Yuting Xu [1], Qun Wang [1,*], Yongchao Sun [1,3], Xiaomeng Wang [1], Jian Wang [4], Jianliang Xue [5] and Xueli Gao [3]

1 College of Chemical and Biological Engineering, Shandong University of Science and Technology, Qingdao 266590, China; skdmaz919@sdust.edu.cn (Z.M.); luluzh5709@163.com (L.Z.); ly19980407@163.com (Y.L.); xyt15153393708@163.com (Y.X.); yongchao_sun@163.com (Y.S.); 15764237673@163.com (X.W.)
2 Sanya Institute of Oceanology, Chinese Academy of Sciences, Sanya 572000, , China
3 Key Laboratory of Marine Chemistry Theory and Technology, Ministry of Education, College of Chemistry and Chemical Engineering, Ocean University of China, Qingdao 266100, China; gxl_ouc@126.com
4 The Institute of Seawater Desalination and Multipurpose Utilization, Ministry of Natural Resources (MNR) Tianjin 300192, China;
swordking8856@163.com
5 College of Safety and Environmental Engineering, Shandong University of Science and Technology, Qingdao 266590, China; ll-1382@163.com
* Correspondence: jixiaoshen@zju.edu.cn (X.J.); wangqun87@sdust.edu.cn (Q.W.)

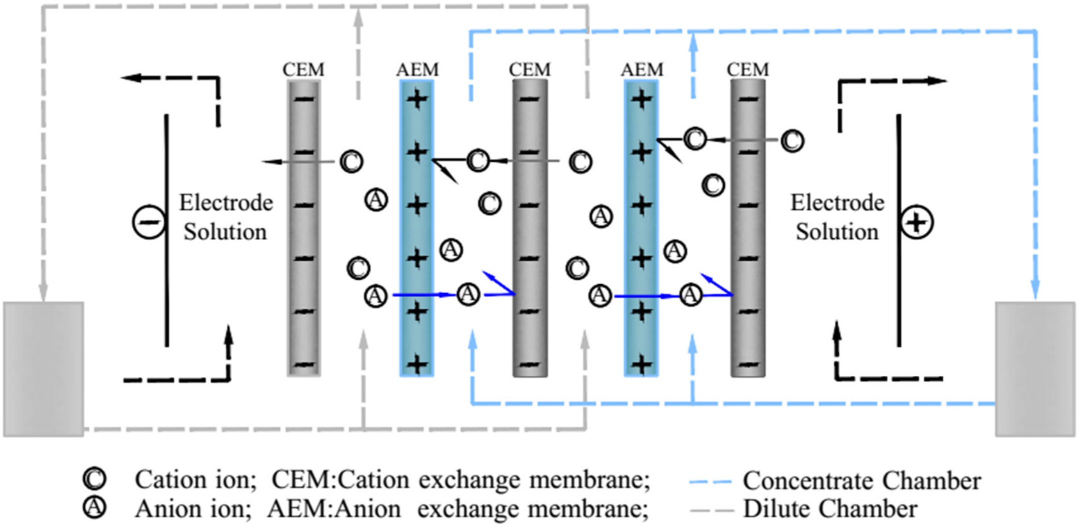

Figure S1. The schematic diagram of the experiment.

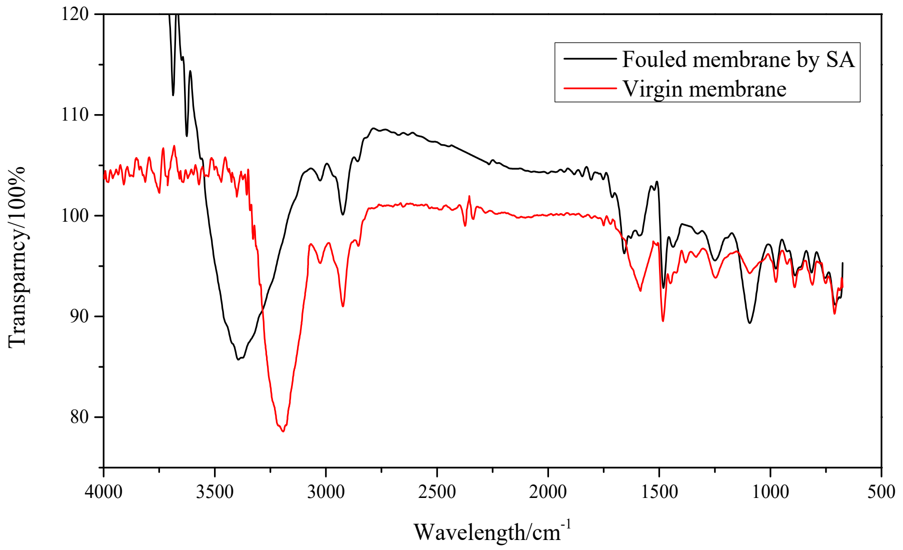

Figure S2. FTIR spectrum of virgin membrane and fouled membrane by SA together with 4 mmol Ca^{2+}.

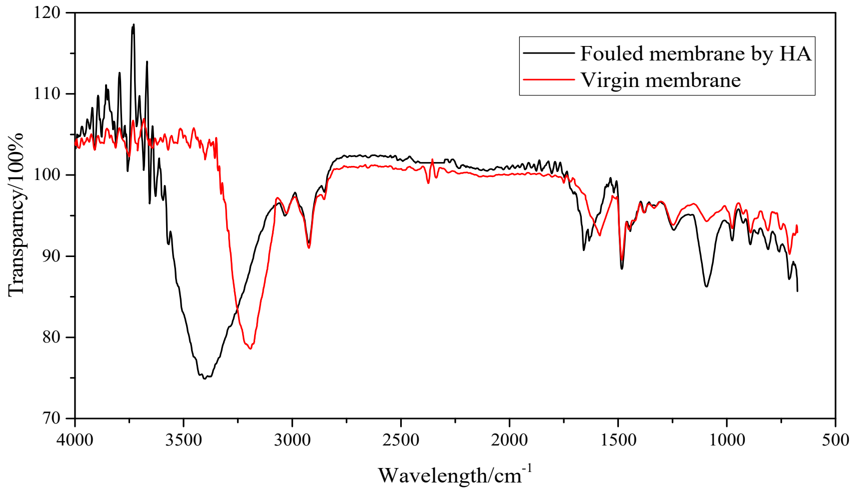

Figure S3. FTIR spectrum of fouled membrane by HA together with 4 mmol Ca^{2+}.

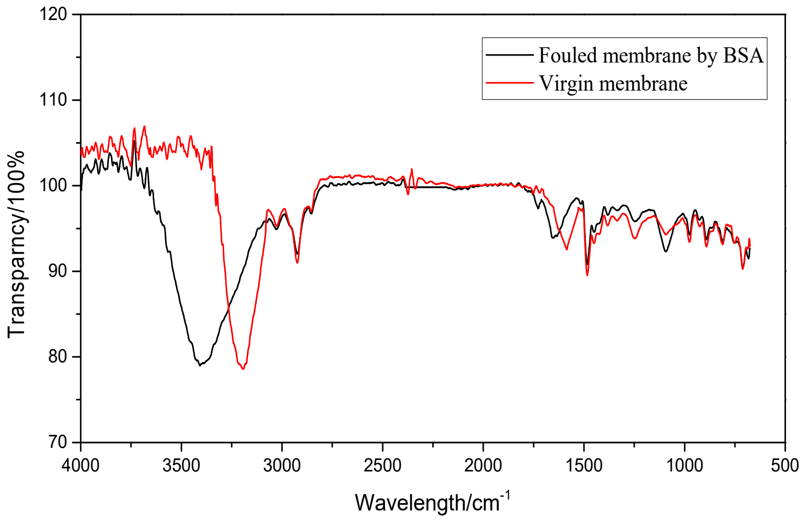

Figure S4. FTIR spectrum of fouled membrane by BSA together with 4 mmol Ca^{2+}.

Table S1. Properties of ion exchange membranes.

Items	Cation Exchange Membrane	Anion Exchange Membrane
Product Name	CMV	AMV
Characteristic	Standard	Standard
Usage	ED	ED
Thickness/μm	120	120
Counterion	Na^+	Cl^-
Burst Strength/kPa	200	200
Resistance*/ Ωcm^2	3.0	2.8
Transport number/%	>96	>96

* Experimental conditions: 25°C and 0.5 mol/L NaCl.

Table S2. Concentration of foulants used in the fouling experiments in ED process .

Organics	Concentrations (mg/L)	Ca^{2+} Concentration (mmol/L)
SA	20	0
		2
		4
		8
HA	20	0
		2
		4
		8
BSA	20	0
		2
		4
		8
